# Association of NK cells with a shift in tryptophan catabolism in patients with heart failure after a single exercise exertion

**DOI:** 10.3389/fimmu.2025.1732461

**Published:** 2026-01-26

**Authors:** Krithika Swaminathan, Bita Astan, Sabine Kaczmarek, Kristin Lehnert, Anke Hannemann, Aycen Koc, Nele Friedrich, Kathrin Budde, Ann-Kristin Henning, Grażyna Domańska, Ulf Landmesser, Christian Templin, Marcus Dörr, Martin Bahls, Nicolle Kränkel

**Affiliations:** 1 Deutsches Herzzentrum der Charité (DHZC) University Hospital Berlin, Department of Cardiology, Angiology and Intensive Care Medicine, Berlin, Germany; 2 DZHK (German Centre for Cardiovascular Research), Partner Site Berlin, Berlin, Germany; 3 Friede Springer - Centre of Cardiovascular Prevention @ Charité, Charité - University Medicine Berlin, Berlin, Germany; 4 Department of Internal Medicine B., University Medicine Greifswald, Greifswald, Germany; 5 DZHK (German Centre for Cardiovascular Research), Partner Site Greifswald, Greifswald, Germany; 6 University Medicine Greifswald, Institute of Clinical Chemistry and Laboratory Medicine, Greifswald, Germany; 7 University Medicine Greifswald, Institute of Microbiology, Greifswald, Germany; 8 University Medicine Greifswald, Institute of Immunology, Greifswald, Germany

**Keywords:** CPET, exercise, HFREF, kynurenine, NK cells, tryptophan

## Abstract

**Background:**

Tryptophan (TRP) metabolism via the kynurenine (KYN) pathway links immune function, energy metabolism, and redox homeostasis. Dysregulation of this pathway has been implicated in inflammatory conditions and heart failure. Here, we investigated the acute effects of exercise on TRP-KYN metabolism and its relationship with natural killer (NK) cell function in controls and patients with heart failure with reduced ejection fraction (HFrEF).

**Methods:**

Control (n=13) and HFrEF (n=16) groups had comparable composition regarding age and sex. Participants were investigated at baseline, immediately after a maximal symptom-limited cardiopulmonary exercise test (CPET), and after 2 hours of resting. Blood samples were obtained at all time points to assess NK cell counts and phenotypic parameters by flow cytometry, as well as tryptophan metabolites and protein secretome by mass spectrometry and targeted proteomics, respectively. NK cells and non-NK cells from blood of healthy donors were stimulated ex vivo prior to flow cytometry-based measurement, indoleamine 2,3-dioxygenase (IDO) mRNA expression analysis and mass spectrometry-based tryptophan metabolite analysis.

**Results:**

Plasma TRP levels decreased post-exercise in both study groups, with increased metabolism down the KYN route, albeit only in HFrEF patients, a significant accumulation of quinolinate (QUIN) was seen. Increases in plasma KYN-to-TRP ratios correlated with more circulating NK cell counts and IL-12p70 levels mainly in the HFrEF group. Ex vivo, IL-12 exposure of human total primary NK cells increased representation of the CD56-bright subset, IDO mRNA expression, and TRP-to-KYN conversion, resulting in net KYN accumulation and elevated QUIN production. In non-NK cells, IFN-γ exposure similarly promoted TRP-to-KYN flux and QUIN formation.

**Conclusion:**

Collectively, our observations confirm earlier descriptive reports of exercise-induced upregulation of KYN production by NK cells and add mechanistic evidence that IL-12 induces a phenotype shift in NK cells, which is accompanied by accelerated TRP metabolism into KYN. Our data point to a concerted interaction between leukocyte subsets upon acute exercise, via the release of IL-12, with potential implications for differential energy metabolism and immune regulation in HFrEF.

## Graphical Abstract

**Figure:**
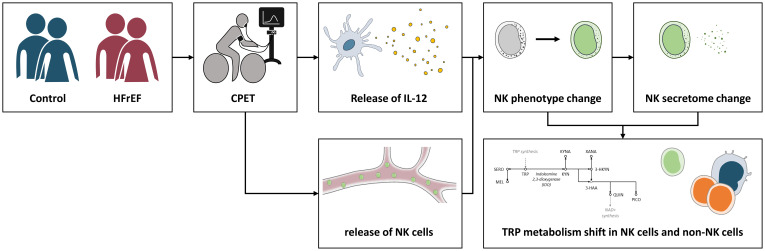

## 1 Introduction

An estimation of about 56.5 million individuals globally live with heart failure (HF). HF contributes substantially to global morbidity and poses a growing burden especially in older populations (1). Cardiopulmonary exercise testing (CPET) facilitates the identification of exercise intolerance in patients with HF, provides prognostic information, and enhances understanding of the different metabolic responses to physical exertion in these patients (2, 3).

The essential amino acid tryptophan (TRP) is metabolized in mammals via three distinct pathways—the kynurenine (KYN), the serotonin and the indole pathway. While the metabolism of dietary tryptophan via the indole route is primarily accomplished by intestinal bacteria, the serotonin and KYN pathways are largely mediated by the host (4). The TRP metabolism via KYN is increasingly recognized for its role in HF pathophysiology (5). Elevated plasma KYN levels and KYN-to-TRP ratios have been associated with reduced functional capacity and adverse outcomes in chronic HF (5). Downstream metabolites of KYN exert differential effects with anti-inflammatory and neuroprotective effects being reported for kynurenic acid (KYNA) (6), while quinolinate (QUIN) appears to contribute to inflammation, oxidative stress and apoptosis (7).

Acute exercise induces rapid changes in TRP metabolism. Studies demonstrate that exercise transiently increases KYN levels, likely mediated by upregulation of indoleamine 2,3-dioxygenase (IDO) during inflammatory or stress states (8). This exercise-induced modulation of TRP metabolism has important implications for immune regulation and neuroprotection (9, 10).

Natural killer (NK) cells, central effectors of the innate immune system, exhibit metabolic plasticity and can influence systemic metabolism (10, 11). Acute exercise has been shown to cause an immediate release of NK cells, to modulate NK cell activity and to influence TRP metabolism via KYN (11–13). Although the effects of exercise on NK cell function and TRP metabolism have been described in healthy individuals, their role in HF patients remains poorly understood. Exercise-induced changes in NK cell activity and TRP metabolism may provide mechanistic insights into disease progression and identify potential therapeutic targets.

In this study, we aimed to characterize acute exercise-induced changes in TRP metabolism in patients with HF with reduced ejection fraction (HFrEF) compared with controls of comparable age and sex. In subsequent *ex vivo* studies, we further investigated mechanistic links between IL-12 stimulation and TRP catabolism in NK cells.

## 2 Materials and methods

### 2.1 Participants and study design

This study constitutes an extension of the MicroEx study (2), where we characterized the overall metabo-inflammatory response to acute exercise in HFrEF. We now specifically examine how natural killer (NK) cells relate to acute exercise-induced alterations in tryptophan–kynurenine metabolism in this population.

HFrEF patients and controls (CON) were recruited at the University Medicine Greifswald. All study visits were scheduled at the same time of day to minimize potential circadian effects. The study was conducted in accordance with the Declaration of Helsinki (2013 revision) and approved by the Institutional Ethics Committee of the University Medicine Greifswald (application no. BB153/17). Written informed consent was obtained from all participants prior to study enrolment. Baseline assessments and blood collection (T1) were performed before participants completed a standardized CPET. A second blood sample was collected immediately post-exercise (T2), followed by a third sample after a two-hour recovery period (T3). Participant characteristics are presented in (**Table 1**).

**Table 1:** Patient characteristics and medication.

Parameter	HFrEF (n=16)	Control (n=13)	p-value
Age (years)	62 ± 10	59 ± 8	0.559
Male (n)	11 (69%)	7 (54%)	0.411
Height (cm)	172 ± 8	172 ± 10	0.746
Weight (kg)	91.2 ± 17.3	85.9 ± 15.7	0.351
BMI (kg/m2)	30.7 ± 5.3	28.9 ± 4.2	0.374
**SBP (mmHg)**	117 ± 16	134 ± 13	**0.008**
**DBP (mmHg)**	67 ± 12	79 ± 8	**0.007**
Creatinin (μmol/l)	83.38 ± 24.44	74.77 ± 9.73	0.531
**HbA1c (%)**	6.4 ± 1.0	5.6 ± 0.5	**0.022**
Lipids			
LDL-c (mmol/l)	2.67 ± 0.99	2.82 ± 0.99	0.398
HDL-c (mmol/l)	1.14 ± 0.25	1.35 ± 0.36	0.075
triglycerides (mmol/l)	2.07 ± 0.76	2.38 ± 1.84	0.714
total cholesterol (mmol/l)	4.15 ± 1.18	4.85 ± 1.33	0.101
Echocardiography			
**LVEF (%)**	34.6 ± 5.6	57.2 ± 5.5	**<0.001**
LAVI (cm2)	33.3 ± 12.8	30.9 ± 8.0	0.914
E/A	1.3 ± 0.9	1.3 ± 0.8	0.531
E/e’	14.5 ± 6.0^a^	10.6 ± 3.7	0.142
**LVEDD (mm)**	63.4 ± 9.2	51.2 ± 7.3	**0.001**
IVSd (mm)	10.9 ± 2.5	10.7 ± 1.4	0.589
PWd (mm)	10.0 ± 1.2	9.6 ± 1.1	0.423
TAPSE (mm)	20.9 ± 4.2^a^	24.2 ± 6.7	0.235
Medication			
**beta blocker**	13 (81%)	6 (46%)	**0.048**
ACE inhibitors	3 (19%)	2 (15%)	0.811
AT-2 receptor blocker	4 (25%)	5 (38%)	0.436
**sacubitril + valsartan**	5 (31%)	0 (0%)	**0.027**
**aldosterone antagonist**	8 (50%)	1 (8%)	**0.014**
diuretics	9 (56%)	4 (31%)	0.17
lipid lowering medication	8 (50%)	4 (31%)	0.296
insulin or other anti-diabetic medication	2 (13%)	1 (8%)	0.672
anti-platelet therapy	4 (25%)	4 (31%)	0.73
**oral anti-coagulant**	5 (31%)	0 (0%)	**0.027**
calcium channel blocker	1 (6%)	2 (15%)	0.422
sodium channel blocker	2 (13%)	0 (0%)	0.186
proton pump inhibitors	3 (19%)	3 (23%)	0.775
NSAID	1 (6%)	2 (15%)	0.422
gout medication	3 (19%)	0 (0%)	0.099
antidepressant	0 (0%)	1 (8%)	0.259
anti-obstructive pulmonary treatment	1 (6%)	0 (0%)	0.359
vitamins/supplements	4 (25%)	1 (8%)	0.220

a n=15 (missing data for 1 participant due to technical defect).Bold values indicate parameters with p<0.05.

For ex vivo NK cell studies, peripheral blood of 3 male and 3 female healthy donors (age: median: 38 years, min: 24 years, max: 53 years; BMI: median: 22.0 kg/m², min: 19.2 kg/m², max: 32.1 kg/m²) was obtained at the Charité – Universitätsmedizin Berlin under a separate protocol approved by the local Ethics Committee (application no. EA2/059/15).

### 2.2 Baseline measurements

During screening, every participant underwent transthoracic echocardiography for assessment of the left and right ventricular function, heart morphology, and valve function. Participant’s general medical history was collected via questionnaire, covering sex, age, socioeconomic status, pre-existing medical conditions, medications, and daily physical activity. Additionally, weight, height, waist circumference, heart rate, electrocardiogram, and blood pressure were measured (**Table 1**).

### 2.3 Symptom-limited cardiopulmonary exercise testing

CPET was performed on a stationary bicycle (MasterScreen CPX system [CareFusion, Höchberg, Germany]) according to the modified Jones protocol (14, 15) under the supervision of trained and certified examiners with on-call doctors, as described before (2).

None of the participants had to terminate exercise prematurely, as no clinically significant abnormalities were observed, including echocardiographic signs of ischemia, arrhythmias, a drop in systolic blood pressure >10 mmHg from baseline, circulatory or respiratory disorders, dyscoordination, confusion, severe dizziness, or hypertensive blood pressure responses. Criteria for achieving peak effort included reaching 90% of the age-predicted maximum heart rate (220 − age), a plateau in the VO_2_ curve, respiratory quotient (RQ) >1.1, or blood lactate >8 mmol/L.

### 2.4 Flow cytometry

Within 30 min of collection, 100 μL EDTA-anticoagulated blood from each time point were stained with directly fluorescence labelled antibodies CD3-PE/Cy7 (cat-no. 300316), CD4-AF488 (cat-no. 317420), CD8-BV510 (cat-no. 301048), CD14-BV421 (cat-no. 301830), CD16-AF647 (cat-no. 302020), CD25-AF700 (cat-no. 302622), CD41-APC/Cy7 (cat-no. 303716), CD45-BV711 (cat-no. 304050) and CD127-BV605 (cat-no. 351334), all BioLegend, San Diego, CA, USA). After 20 minutes of incubation, samples were fixed/diluted with 1% paraformaldehyde in phosphate-buffered saline (PBS). Samples were shipped at 4 °C and measured in an Attune NxT Acoustic Focusing Cytometer (Thermo Fisher Scientific) within 36h. The data compensation was performed according to standard procedures prior to analysis. Acquired.fcs files were analyzed in Kaluza (Analysis version 2.1) as described before (16).

### 2.5 Cytokine profiling

A total of twenty cytokines were determined using the Inflammation 20-Plex Human ProcartaPlex™ Panel (Thermo Fisher Scientific) in cleared EDTA plasma according to the manufacturer’s instructions.

### 2.6 Metabolomics

Acid Citrate Dextrose (ACD)-anticoagulated blood samples were centrifuged immediately after collection and stored at -80°C. Targeted metabolomics profiling of the plasma samples was performed using the MxP^®^ Quant 500 Kit (BIOCRATES LifeSciences AG, Innsbruck, Austria) as recommended by the manufacturer and TRP metabolites were assessed using the method described by Fuertig et al. (17) with modification as delineated in the online supplement.

### 2.7 Natural killer cell isolation, culture and stimulation

NK cells were isolated from peripheral blood mononuclear cells (PBMC) of healthy donors by negative depletion using the human NK cell isolation kit (Miltenyi Biotec, Bergisch Gladbach, Germany). Seven to eight x 10^4 purified NK cells of each donor were cultured per mL per well of a 12-well plate for 2 days in RPMI medium supplemented with glutamine, 10% fetal bovine serum (FBS) and penicillin (100 U/mL)-streptomycin (100 μg/mL).

In order to study NK cell phenotype shift, freshly isolated NK cells from the same donor were seeded into 2 parallel wells and interleukin (IL)-12 (final concentration 10 ng/mL) was added to the medium of the IL-12 group, while the control group was cultured without additional additives, as described above. At the end of day 2, the cells were harvested and the cell-free supernatants were collected for further experiments.

### 2.8 Flow cytometry of isolated NK cells

Cells were stained with fluorochrome-conjugated monoclonal antibodies targeting surface markers to distinguish NK cell subsets: CD3-AF488 (cat-no. 300320), CD14-Pacific Blue (cat-no. 301828), CD16-AF647 (cat-no. 302020), CD45-BV711 (cat-no. 304050) and CD56-BV510 (cat-no.318340), all from BioLegend, San Diego, CA, USA and Sytox (cat-no. S34860, Thermo fisher Scientific). For surface staining, 100 µL of cell suspension was incubated with antibodies for 30 minutes at room temperature in the dark. Cells were then washed with PBS and fixed/diluted with 0.5% paraformaldehyde in PBS. Flow cytometric acquisition was performed using Attune NxT Acoustic Focusing Cytometer (Thermo Fisher Scientific). Flow cytometry data was compensated using standard methodologies prior to analysis to correct for spectral overlap between fluorochromes. Acquired.fcs files were analyzed in Kaluza (Analysis version 2.1). At least 50,000 events were recorded per sample.

### 2.9 Experimental setup for NK cells – TRP metabolomics

PBMCs were isolated from 30 mL of EDTA-anticoagulated blood from six healthy volunteers, after which NK cells were purified by negative selection using the Miltenyi Biotec human NK Cell Isolation Kit. Labeled cells (i.e. non-NK cells) were retained in the magnetic column, while the flow-through contained unlabeled cells (enriched NK cells) and was collected as the “NK cell fraction”. The non-NK cell fraction, consisting predominantly of monocytes and other lymphocytes, was collected separately by subsequential elution after separating the column from the magnet. A total of 5 × 10^5 cells from each fraction (NK cells or non-NK cells) were plated per well in two separate 48-well plates, allowing duplicates for each condition, donor, and cell fraction. NK cells were exposed to IL-12 (10 ng/ml) or left untreated and non-NK cell fraction was exposed to IFN-γ (20 ng/ml). Samples were collected after 4 h from plate 1 and after 24 h of incubation from plate 2. Throughout all experiments, cells were cultured in RPMI as before, but with the addition of L-tryptophan at a final concentration of 5 µM. Unconditioned medium and freshly isolated cells at 0 h were frozen as baseline controls. A graphical overview of the experimental setup is provided in the online supplement.

At each harvest time point, cells and medium were collected and separated by centrifugation (300 RCF, 8 minutes, 4°C). Cells were sonicated, incubated with protease inhibitor (cOmplete™, Merck, cat-no. 04693132001) and centrifuged at 16000 RCF for 15 minutes at 4°C to pellet debris. Cleared supernatant as well as cell-conditioned medium were aliquoted separately and stored at −80°C until measurement. Supernatants and sonicated cells were used for assessment of tryptophan metabolites by mass spectrometry as delineated in the online supplement.

### 2.10 Analysis of IDO mRNA expression in NK cells

Total RNA was extracted from purified NK cells using the miRNeasy Micro Kit (cat-no. 1071023, Qiagen) according to the manufacturer’s instructions. RNA concentration and purity were assessed using a Nanovue plus spectrophotometer (GE healthcare). Complementary DNA (cDNA) was synthesized from 200 ng of total RNA using the High-capacity cDNA reverse transcription Kit (cat-no. 4368814, Applied biosystems, Thermo Fisher Scientific) following the manufacturer’s protocol. Quantitative real-time PCR (qPCR) was performed using the SYBR Select Master Mix (cat-no. 4472908, Applied biosystems, Thermo Fisher Scientific) on a Viia7 Real-Time PCR Detection System (Applied Biosciences). PCR reactions were run in a final volume of 10 μL containing 5 μL SYBR Select Master Mix, 0.5 μM of each primer, and 1 μL cDNA. Relative IDO1 mRNA expression was calculated using the 2^−ΔΔCt method, normalized to GAPDH, and expressed as fold change relative to the unstimulated control condition. All samples were measured in technical triplicates.

### 2.11 Statistical analysis

Data were compiled in Excel 2019 and analyzed using R version 4.5.2 (R Foundation for Statistical Computing, Vienna, Austria). Parameters were eliminated for all participants when they were biologically or biochemically redundant, or when more than 10% of values were missing. For all the replicate measurements, median was used for analysis.

Several parameters showed non-normal distributions of the within-subject differences, and sample sizes were small. Therefore, nonparametric methods were chosen throughout. HFrEF and control groups were assessed separately.

Paired Wilcoxon Signed-Rank test was used to compare between two groups of stimulated and unstimulated cells originating from the same donor and isolation batch.

For all comparisons between time points T1 and T2 (assessing changes upon acute exercise/CPET) and between T1 and T3 (assessing recovery), values of the same individuals were compared and therefore paired analyses were employed.

Spearman rho correlations were performed in HFrEF and control groups separately, using the calculated ratios between T2 and T1 to assess acute response to CPET. The Benjamini-Hochberg method was applied to adjust for multiple testing.

An alpha level of 0.05 was applied throughout.

## 3 Results

### 3.1 Acute exercise enhances TRP-KYN conversion and downstream metabolite accumulation

**Figure 1:**
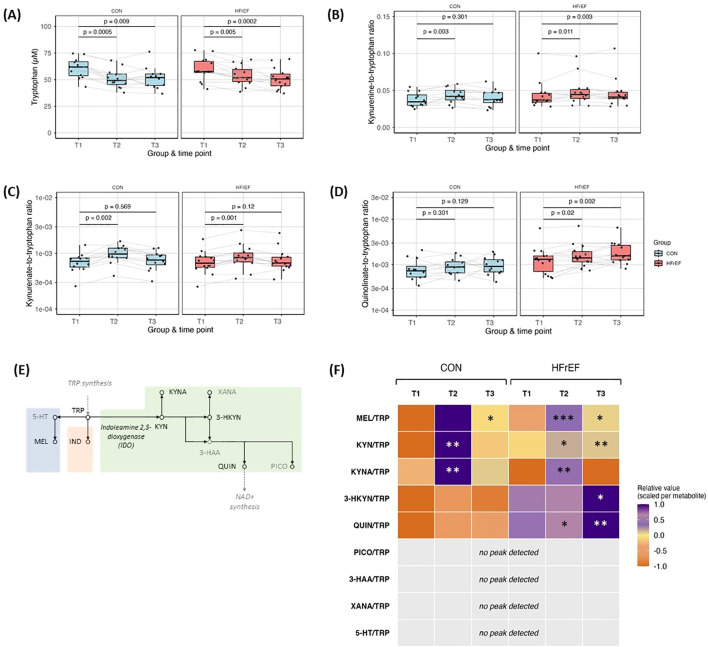
Plasma levels of amino acid tryptophan were reduced after acute exercise in controls and HFrEF **(A)**. The KYN-to-TRP ratio rose acutely in both groups **(B)**. Synthesis of kynurenate increases acutely in both groups **(C)**, while further metabolization into quinolinate **(D)** is delayed and more pronounced in the HFrEF group. ​Schematic overview of TRP metabolism **(E**, adapted from the KEGG database**)** (18, 19). Heatmap representing mass-spectrometry analysis of TRP metabolites showing elevated flux towards SERO route and KYN route **(F)**. Heatmap scaling was done per metabolite, each value was linearly mapped into [-1, 1], symmetric around the metabolite median where -1 is minimum, 0 is median and 1 is maximum. P values are indicated for T2 vs T1 (CON and HFrEF) and T3 vs T1 (CON and HFrEF): *** < 0.001** < 0.01; * < 0.05.

Plasma TRP concentrations were significantly reduced following acute exercise in both control and HFrEF groups (**Figure 1A**). TRP serves as a substrate in multiple metabolic pathways, including those related to serotonin and melatonin biosynthesis, as well as the kynurenine pathway (**Figure 1E****-** adapted from KEGG: map00380, Tryptophan metabolism) (18, 19). Mass spectrometry–based analysis of additional TRP metabolites indicates elevated flux down the serotonin (5-HT) and KYN (**Figure 1B**) routes in response to acute exercise, with a greater flux down the KYNA (**Figure 1C**) route as compared to the serotonin route and differences between controls and HFrEF patients in the utilization of the KYNA versus 3-hydroxy kynurenine (3-HKYN) to QUIN routes (**Figures 1D, F**). TRP to KYN and subsequently to kynurenate acutely increased at T2 in both patient groups, with accumulation of 3-hydroxykynurenine and QUIN observed at T3 in the HFrEF group (**Figures 1B, D, F**; **Supplementary Figure 1A**). Furthermore, the melatonin-to-TRP ratio increased at T3 in both groups (**Supplementary Figure 1B**). Additionally, indoxyl sulphate, indole-3-acetic acid in the indole pathway increased at T2 in both groups (**Supplementary Figures 1C–E**).

### 3.2 IL12 drives NK cells towards regulatory phenotype

**Figure 2:**
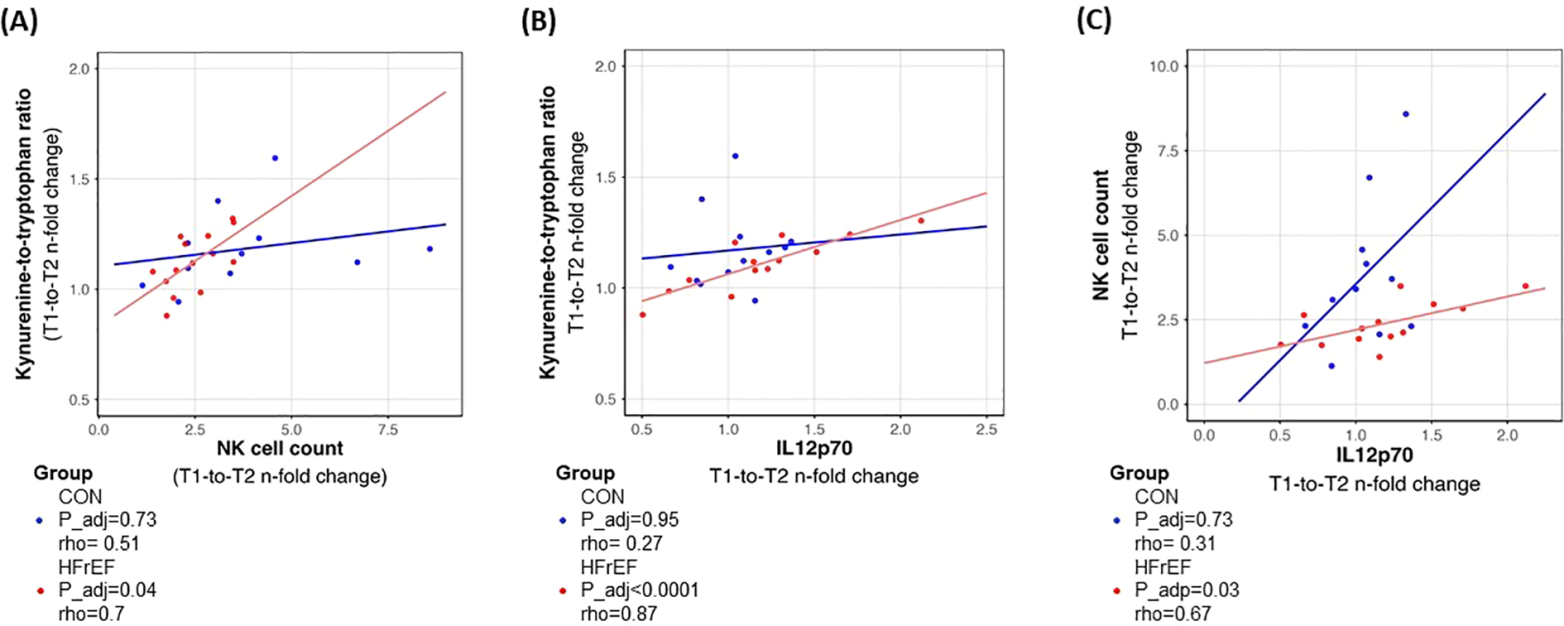
Acute increase in KYN-to-TRP ratio is correlated to acute changes in NK cell count **(A)**. KYN-to-TRP ratio also correlated to the changes in plasma IL12p70 levels in HFrEF **(B)**. Changes in the NK cell counts in the circulation correlated with the changes in the plasma IL12 levels after acute exercise in the HFrEF **(C)**.

Shifts in the counts and activation of circulating NK cells and other leukocyte subsets following acute exercise have been proposed to drive the observed shift in TRP downstream metabolic flow (20), but mechanistic validation has not been performed. We performed correlation analyses between the plasma Kyn/Trp ratio and the circulating number of all major leukocyte populations (**Supplementary Table 1**) and a panel of 19 cytokines (<10% missing values/below detection limit were present for IL-13 and the parameter was therefore not used.) (**Supplementary Table 2**), as well as between the increase of circulating NK cell counts and the cytokine panel (**Supplementary Table 3**). Results highlight associated increases in circulating NK and NK-T cell counts and KYN-to-TRP ratio between T1 and T2 in HFrEF patients but not in controls (**Figure 2A**, **Supplementary Figure 3A**, **Supplementary Table 1**). In HFrEF plasma only, change of both, KYN-to-TRP ratio as well as circulating NK cell count, between T1 and T2 correlated with IL-12p70 and IL-1β (**Figures 2B, C**, **Supplementary Tables 2**, **3**). Due to the stronger association, IL-12p70 was selected for subsequent *ex vivo* experiments.

**Figure 3:**
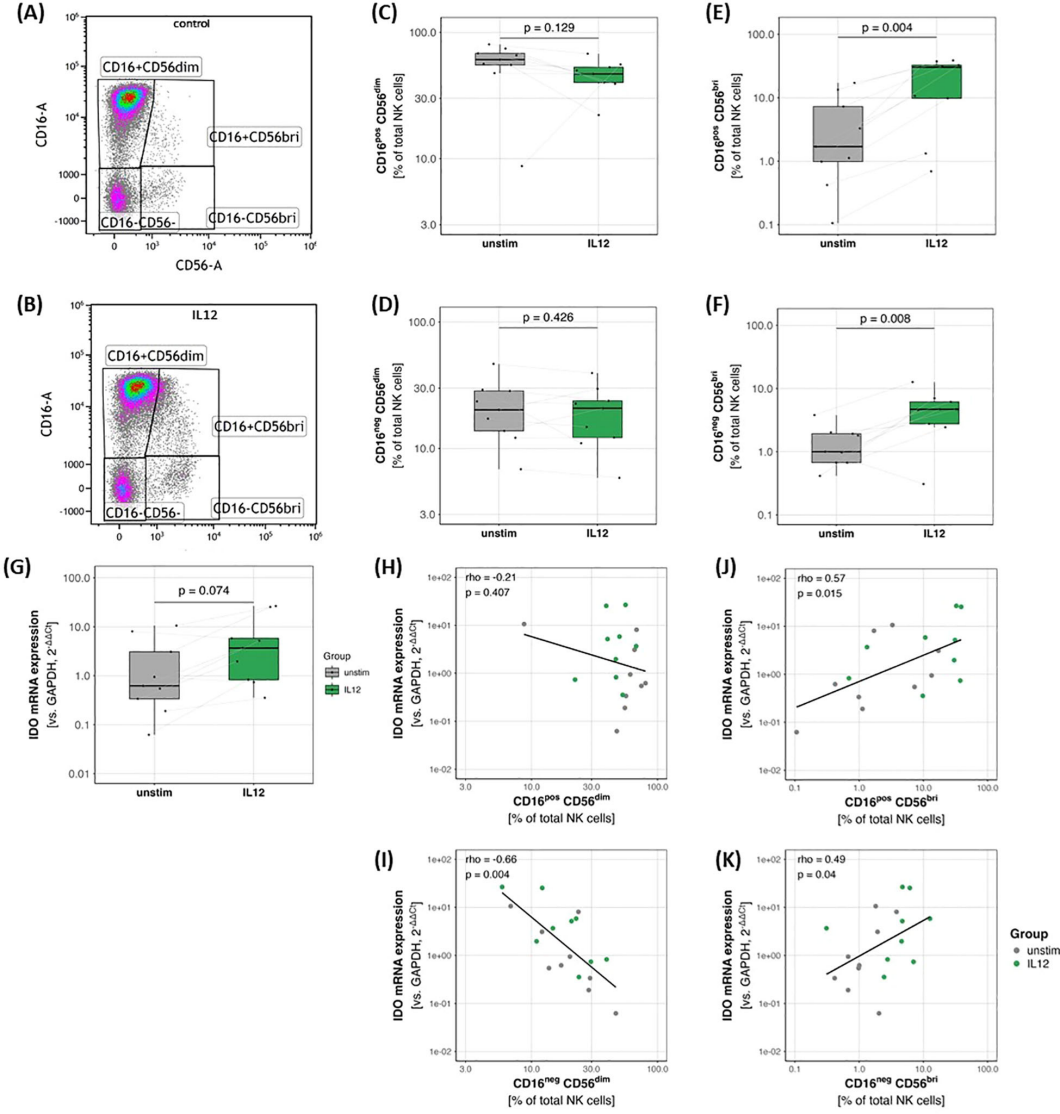
Flow cytometric characterization of NK cell phenotypes after 2 days of culture in the absence **(A)** or presence of IL-12 **(B)**. IL-12 drives NK cell phenotype towards CD56bright immunoregulatory phenotype **(B, E, F)**, while no significant differences were observed for the CD56^dim^ NK cell populations **(C, D)**. Transcription of IDO tended to be higher in IL-12 exposed NK cells than in control conditions **(G)**, correlating with the percentage of CD56^bri^ NK cell phenotypes within the same cultures **(J, K)**. CD16^neg^CD56^dim^ NK representation among total NK cells inversely correlated with IDO mRNA expression **(I)**, while no significant association was observed for CD16^pos^CD56^dim^ NK cells **(H)**.

Exposure of NK cells isolated from healthy human donors to recombinant IL-12 led to an increased relative abundance of CD56 bright (CD56^bri^) NK cell subsets (**Figures 3A, B, E, F**) and no significant change in the CD56^dim^ NK cells (**Figures 3A–D**). IL-12 treatment did not affect cell death rates across NK cell phenotypes (**Supplementary Figures 4A–D**).

### 3.3 IL12 induced NK cell phenotype shift is associated with enhanced TRP-KYN metabolism

NK cells exposed to IL-12 tended to express higher levels of indoleamine 2,3-dioxygenase (IDO) mRNA (p = 0.074; **Figure 3G**) and IDO mRNA levels showed a positive association with the relative abundance of CD56^bri^ (**Figures 3J, K**) and a negative association with CD16^neg^CD56^dim^ NK cells (**Figure 3I**), while no significant association was observed for CD16_pos_CD56_dim_ NK cells (**Figure 3H**).

**Figure 4:**
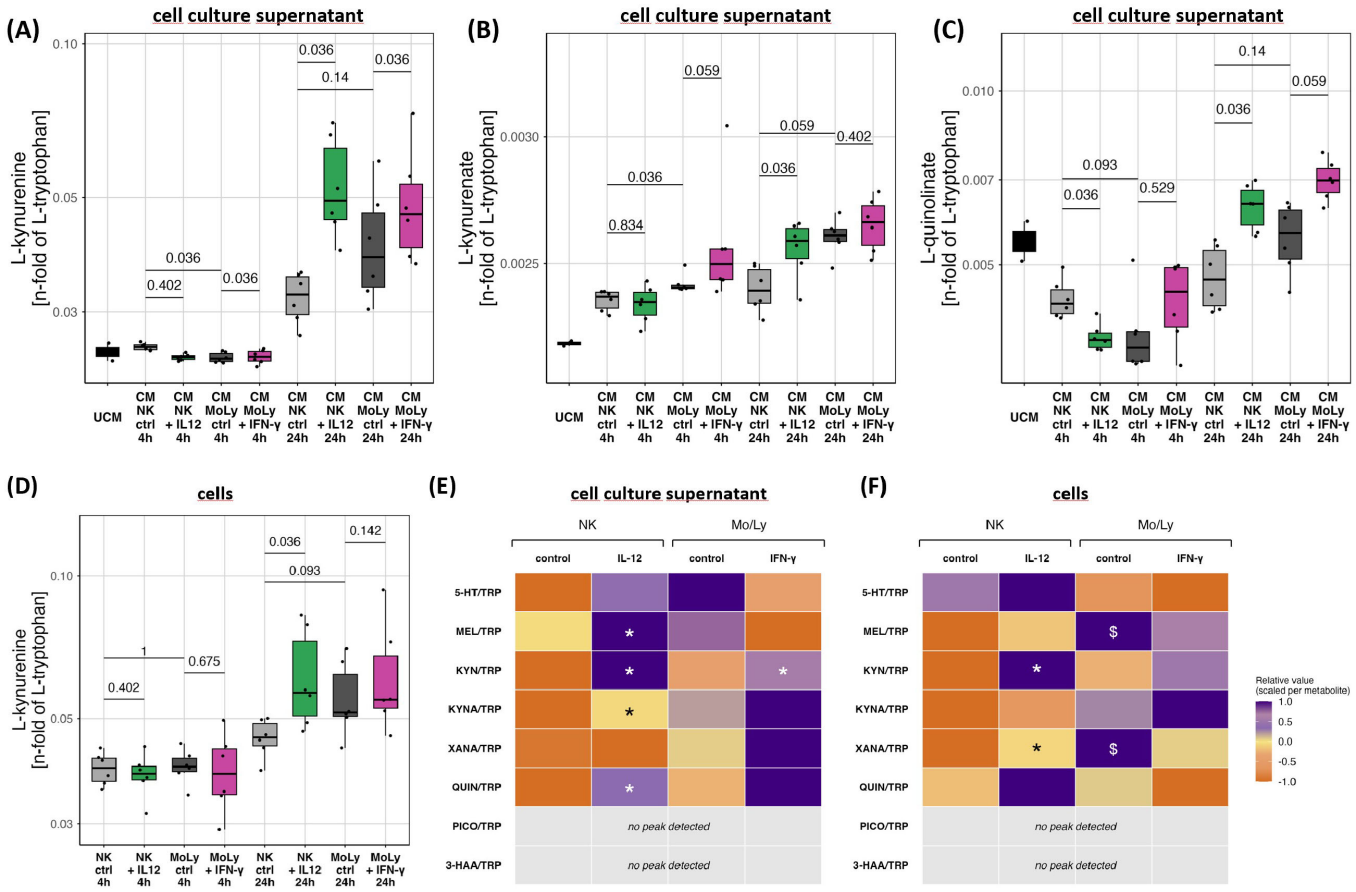
Exposure to IL-12 significantly increases release of KYN **(A, D)**, KYNA **(B)** and QUIN **(C)** from IL-12 treated NK cells after 24h. IFN-γ exposure increases KYN release **(A)** and tends to increase QUIN **(C)** release by Mo/Ly. Heatmaps summarizing overall tested tryptophan metabolites in supernatant **(E)** and cells **(F)**. Heatmap scaling was done per metabolite, each value is linearly mapped into [-1, 1], symmetric around the metabolite median where -1 is minimum, 0 is median and 1 is maximum. P values are indicated for comparison between treated cells versus their respective untreated controls: * < 0.05, and for comparison between untreated NK cell controls and untreated Mo/Ly controls: $ < 0.05.

Concomitantly, NK cells exposed to IL-12 metabolized TRP to KYN at a higher rate than unstimulated NK cells and release it into the supernatant at 24 h, but not at 4h, resulting in net KYN accumulation over time (**Figures 4A, D, E, F**). Consistently, KYNA/TRP and QUIN/TRP ratios in the supernatant are increased in the IL-12 group versus the unstimulated NK group at 24 h, but not at 4 h (**Figures 4B, C**).

Beyond their cytotoxic effector functions, NK cells, particularly CD56^bri^ subsets, also exert regulatory roles, including cytokine release such as interferon (IFN)-γ (21). Upon exposure to IFN-γ, KYN/TRP ratio in the supernatant of the non-NK cell fraction of peripheral blood mononuclear cells (PBMCs), predominantly lymphocytes and monocytes, increased at 24 h (**Figure 4A**). At 24 h, the QUIN/TRP ratio within non-NK cell supernatant tended to increase with IFN-γ stimulation (p = 0.059; **Figure 4C**). The unstimulated non-NK cell fraction converted TRP to xanthurenate (XANA) and melatonin at a higher rate than unstimulated NK cells of the same donor (**Figure 4F**, **Supplementary Figures 7C, E**).

## 4 Discussion

We report an acute reduction of tryptophan plasma levels and further metabolization down the KYN pathway upon a single exhaustive exercise session in control subjects and HFrEF patients, albeit metabolic routes downstream of KYN appear to differ between studied groups.

A single session of endurance exercise has been reported previously to activate the kynurenine pathway, increasing KYN/TRP ratio and downstream metabolites in healthy individuals (20). Participant´s metabolic status and the presence of metabolic dysfunction, as well as physical fitness, exercise intensity and duration might modulate KYN-pathway responses to acute exercise (22). Skeletal muscle–driven metabolism plays an important role in this process: Peroxisome proliferator-activated receptor gamma coactivator-1 alpha 1 (PGC-1α1) activation in skeletal muscle upregulates kynurenine aminotransferases (KAT), which convert KYN into KYNA and thereby lower the pool of KYN available for conversion into downstream metabolites such as 3-HKYN and QUIN (23). In mice, stress-induced depression is associated with KYN → KYNA rerouting, and muscle-specific overexpression of PGC-1α1 lowers KYNA levels and confers resilience to stress (23, 24). In humans, increased muscle KAT expression together with elevated plasma KYNA have been reported after sustained exercise training (22, 25).

The distinction between a single acute exertion and long-term endurance training is central to interpreting our data. Repetitive endurance training promotes sustained upregulation of muscle PGC-1α and KATs, which favors KYNA production and may exert longer-term anti-inflammatory and neuroprotective effects (23). By contrast, a single exhaustive bout (as in our CPET) triggers immediate stress and immune activation, which can transiently upregulate IDO in immune cells and shift TRP flux towards KYN formation; tissue-specific enzyme activity (muscle KATs vs immune IDO/liver TDO) and the timing of sampling determines whether KYNA or QUIN predominates in the circulation after exercise (22). We have previously reported lower maximal performance (Watt) and a lower body weight-normalized peak oxygen uptake, shorter duration of cycling until exhaustion in HFrEF participants than in controls of our study (2). Thus, our observation of a greater acute KYNA response in controls versus increased QUIN accumulation in HFrEF during recovery is consistent with a scenario in which patients with HFrEF may have greater immune-driven IDO activity and/or lower adaptive KAT responses that favors QUIN production, while controls can still mount rapid conversion of KYN to KYNA (muscle KAT activity and/or rapid clearance) (22–24).

Metabolic health also shapes pathway flexibility: Acute exercise-induced TRP → KYN → KYNA shifts can be quantitatively altered in individuals with impaired glucose tolerance or type-2 diabetes, where baseline KAT expression may be lower and metabolic comorbidity may limit full adaptive rerouting (26). In our cohort, HFrEF patients displayed a higher HbA1C, indicative of impaired glucose metabolism compared with controls (2), that may reduce muscle PGC-1α/KAT responsiveness and favor immune-driven IDO activity; this might provide an explanation for the relatively greater QUIN accumulation during recovery in HFrEF. Taken together, acute exercise-induced shifts in TRP metabolism as seen in our study, are likely modulated by the intrinsic capacity for muscle-mediated rerouting (fitness/training, muscle PGC-1α/KAT) and the magnitude of acute stress biology (immune activation/IDO, comorbidity). Our observations can therefore not be allocated precisely to a single pathological entity; rather, they reflect the typical multimorbidity phenotype of clinical HFrEF patients and underscore the need for mechanistic interventional trials (e.g., endurance training, metabolic risk reduction) and tissue-level measurements (muscle KAT expression, leukocyte IDO activity, NAD^+^ flux) to establish causality (22, 25, 27).

Typically, NK cells are acutely released upon exercise and are associated with shifts in tryptophan metabolites in young and healthy athletes (20). Joisten et al. further demonstrated that changes in plasma KYN and KYN-to-TRP ratio correlate with IL-6 levels and CD56^bri^ NK cell abundance, alongside increased leukocyte IDO1 expression after acute exercise (20). While previous studies have reported specific processes such as the exercise-induced release of cytokines (9) and activation of leukocytes, especially NK cells (20), our data strengthen this connection between immune regulation and energy metabolism. This connection is exemplified by the role of NK cells in contributing to post-exercise TRP metabolism.

Our cell culture data confirm the prior associations and add mechanistic evidence that IL-12 induces a phenotype shift in NK cells obtained from healthy donors *ex vivo*, which is accompanied by accelerated TRP metabolism into KYN and further towards QUIN, mirroring our observations in HFrEF plasma following CPET. While previous work has described that acute exercise transiently mobilizes NK cells, with a relative enrichment of CD56^bri^ subsets during recovery (28), we show that IL-12 may represent a cytokine driver of this shift. In the non-NK cell fraction, treatment of IFN-γ, the prototypical messenger of CD56^bri^ NK cells, led to a shift in TRP metabolism away from serotonin/melatonin towards metabolites further downstream of kynurenine, namely KYNA, xanthurenate and QUIN. These findings affirm NK cells as an additional immune source of KYN metabolites under cytokine stimulation, advancing prior associative observations (23), and suggest a concerted interaction between leukocyte subsets upon acute exercise, involving cytokine players such as IL-12 and IFN-γ.

A potential anti-inflammatory role of KYN metabolites in modulating immune responses and influencing disease outcomes has been reported (6). In our study, the post-exercise increase in the KYN-to-TRP ratio indicates enhanced flux through the KYN pathway, also promoting production of KYNA, which might potentially exert anti-inflammatory effects and was more pronounced in the control group. The accumulation of 3-HKYN and QUIN in HFrEF after the resting/recovery period may reflect disease-related *in vivo* microenvironmental regulation [e.g. impaired mitochondrial function (29)] as well as reduced KAT availability and may potentially limit exercise-induced anti-inflammatory effects of TRP-KYN metabolism. QUIN serves as a precursor for NAD+ biosynthesis, a critical cofactor for energy metabolism, sirtuin activity, and DNA repair (30, 31). Previous studies have reported lower myocardial nicotinamide adenine dinucleotide (NAD+) levels in heart failure (32, 33). Our data might therefore suggest an impaired QUIN metabolization down the NAD+ *de novo* synthesis route. However, this remains speculative since other NAD+ synthesis pathways (Preiss–Handler pathway, Salvage pathway (12)) might be utilized by cells to synthesize NAD+ and we have not examined NAD+ synthesis in this study. Functionally, one consequence of QUIN accumulation in HFrEF might be greater levels of oxidative stress, inflammation, and tissue injury, as suggested by findings of kidney samples (7).

Taken together, the exercise-induced release of NK cells might be part of a concerted activation of inflammation-resolving and tissue repair processes, also including re-routing of TRP metabolism (via KYN/KYNA) in control individuals. Exposure to increased plasma levels of IL-12 and potentially other cytokines post-exercise in HFrEF might aggravate pro-inflammatory processes via a shift of KYN metabolism towards QUIN.

When interpreting our observations, several limitations need to be considered: First, our sample size was relatively small, due to greater logistical and methodical efforts of the CPET. Second, our study sample consisted of Caucasian individuals only and our results may not be generalizable to other ethnicities. Our study design does not allow disentangling the relative contributions of muscle-derived versus immune cell–derived KYN metabolism. We did not measure muscle KAT expression or tissue NAD^+^ in this study; therefore, the inferred muscle contribution to KYNA production and the interpretation of QUIN as NAD^+^ precursor remain hypotheses that require direct tissue measurements. Without direct assessment of the molecular players (KATs, IDO/TDO (liver)) within the relevant tissues/cell types (e.g. muscle biopsies, freshly isolated NK cells, (liver)) before and after exercise and inhibitory approaches, we cannot definitively assign the source of circulating metabolites. The presence of metabolic risk factors (e.g. HbA1c) in HFrEF patients constitutes a confounder and limits attribution of findings solely to heart failure pathophysiology. Beta blockers and, as is to be expected, guideline recommended medication for HFrEF (ARNi, aldosterone antagonists), differ between groups but cannot be stopped/interrupted for study purposes due to ethical reasons. Medication might therefore constitute a confounding factor.

Exercise challenge-based metabo-inflammatory phenotyping may allow early detection of metabolic dysregulation during disease onset and/or a better resolution of disease phenotype. Future interventional studies might help to better understand the modifying role of individual risk factors (HbA1c, physical fitness/performance status) on exercise-induced re-routing of TRP metabolism and test whether tailored exercise strategies can restore protective TRP→KYN→KYNA metabolization. Mechanistic analyses might incorporate high-resolution metabolite sampling and assess cell- and tissue-level enzyme expression before and after exercise challenge to disentangle immune-versus-peripheral contributions to TRP catabolism and potential subsequent consequences on energy metabolism and organ damage/protection.

## Data Availability

The raw data supporting the conclusions of this article will be made available by the authors, without undue reservation.
