# Supplementary material for: Association of NK cells with a shift in tryptophan catabolism in patients with heart failure after a single exercise exertion
Source: Front Immunol. 2026 Jan 26;16:1732461. doi: 10.3389/fimmu.2025.1732461 (PMC12883804; doi:10.3389/fimmu.2025.1732461)
Supplement: Supplementary file 1 [file DataSheet1.docx]

***Supplemental Information***

Association of NK cells with a shift in tryptophan catabolism
in patients with heart failure after a single exercise exertion

## Krithika Swaminathan1,2,3, Bita Astan3, Sabine Kaczmarek4,5, Kristin Lehnert4,5, Anke Hannemann5,6, Aycen Koc1,2, Nele Friedrich5,6, Kathrin Budde5,6, Ann-Kristin Henning5,7, Grażyna Domańska8, Ulf Landmesser1,2,3, Christian Templin4,5, Marcus Dörr4,5, Martin Bahls4,5, Nicolle Kränkel1,2,3*

^1^ Deutsches Herzzentrum der Charité (DHZC) University Hospital Berlin, Department of Cardiology, Angiology and Intensive Care Medicine, Campus Benjamin Franklin, Hindenburgdamm 30, 12203 Berlin, Germany

^2^ DZHK (German Centre for Cardiovascular Research), Partner site Berlin, Germany

^3^ Friede Springer - Centre of Cardiovascular Prevention @ Charité, Charité - University Medicine Berlin, Berlin, Germany

^4^ University Medicine Greifswald, Department of Internal Medicine B, Greifswald, Germany

^5^ DZHK (German Centre for Cardiovascular Research), partner site Greifswald, Germany

^6^ University Medicine Greifswald, Institute of Clinical Chemistry and Laboratory Medicine, Greifswald, Germany

^7^ Institute of Microbiology, University of Greifswald, Greifswald, Germany

^8^ Institute of Immunology, University Medicine Greifswald, Greifswald, Germany

*** Correspondence:**

Nicolle Kränkel, PD Dr. rer. nat., Dipl.-Ing. (FH) Charité – Universitätsmedizin Berlin

Campus Benjamin Franklin, Dept. of Cardiology Hindenburgdamm 30, 12203 Berlin, Germany

phone: +49-(0)30-450-522256

email: [nicolle.kraenkel@dhzc-charite.de](mailto:nicolle.kraenkel@dhzc-charite.de)

# Supplemental Methodology

## Analysis of Tryptophan and its metabolites by mass spectrometry

*Cell culture and stimulation experimental setup:*


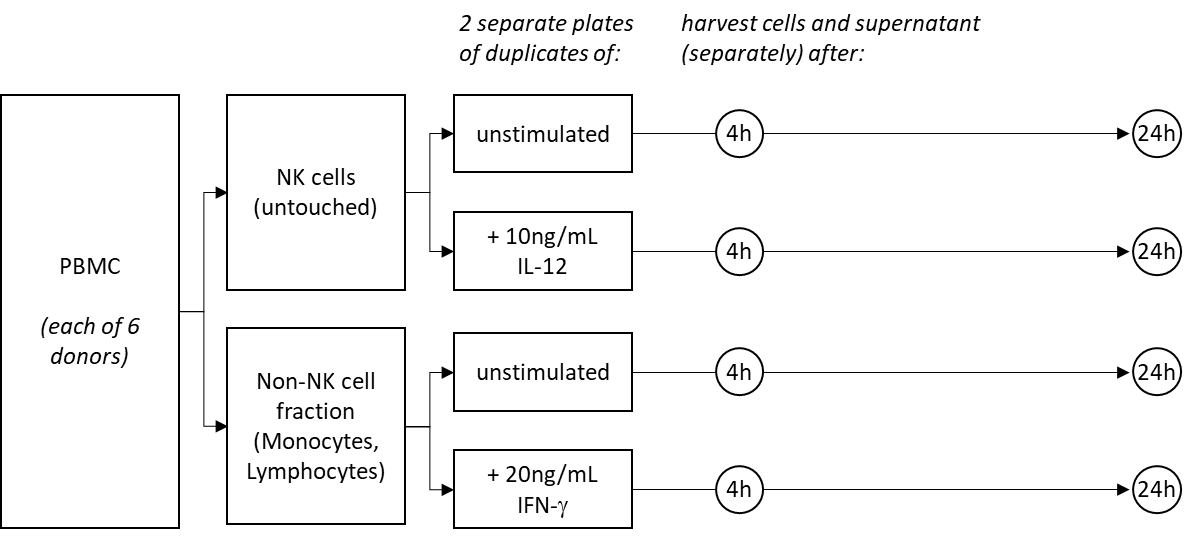
Please refer to the main document for description of media and cell isolation.

*Composition of the calibration standards:*

Starting from 1mmol/L stock solutions of alle standards, 7 different calibration standard dilution mixes (Cal1 to Cal7) in a range from 1 to 204800 nmol/L were prepared. The dilution took place with an acidified mobile phase (0.4% formic acid, 1% acetonitrile in water).

*Preparation of samples and calibration standards:*

10 µl of EDTA-blood plasma or 10 µl of calibration standard was mixed with 10 µl of an internal standard mix (10 µM D5-kynurenic acid, 10 µM D5-phenylalanine, 5 µM D6-kynurenine, 10 µM D5- tryptophan, 5,5 nM 15N5-8-hydroxy-2-deoxyguanosine) and with 10 µl of acidified mobile phase (0.4% formic acid, 1% acetonitrile in water). Mixture was gently shaken on a mixer and 150 µl of ice-cold methanol was added. Samples were incubated overnight at -20°C to allow protein precipitation. On the following day, samples were centrifuged at 0°C and 18 000 g for 15 min. Supernatants were transferred to a new Eppendorf and the liquid phase was removed by evaporation at 30°C and under vacuum. Solid samples were stored until measurement at -20°C. Directly before measurement, dried extracts were reconstituted in 100 µl of acidified mobile phase. Samples were incubated at 40°C for one hour and then centrifuged at 4°C and 18 000 g for 5 min. Clear supernatant (100 µl) were transferred onto a 96-well plate.

*Liquid chromatography tandem mass spectrometry (LC-MS/MS):*

The measurements were performed on an AB Sciex 5500 QTrap™ mass spectrometer (AB SCIEX, Darmstadt, Germany) with electrospray ionization in positive mode combined with a HPLC system (Agilent 1260 Infinity Binary LC, Santa Clara, United States) including a degasser unit, column oven, autosampler and a binary pump. Twenty microliters of the supernatant was injected and separated by using a VisionHT C18 column (100x2.1 mm, particle size 3 µm; Grace, Maryland, United States). In order to prevent contamination of the analytical column a precolumn of the same column material (VisionHT C18, Guard 5x2mm; Grace, Maryland, United States) was used additionally.

The temperature of the column oven was set at 15 °C. The flow rate was set to 0,4 ml/min and the sample was separated in a total run time of 11 minutes using solution A (Water + 0,1 % FA + 0,01 % TFA) and solution B (MeOH + 0,1 %FA + 0,01 %TFA) with the following gradient (Table 1):

| **Total Time [min]** | **Flow Rate [µl/min]** | **Solvent A [%]** | **Solvent B [%]** |
| --- | --- | --- | --- |
| **0.00** | 400 | 97 | 3 |
| **2.80** | 400 | 97 | 3 |
| **3.30** | 400 | 70 | 30 |
| **4.30** | 400 | 40 | 60 |
| **5.00** | 400 | 40 | 60 |
| **5.50** | 400 | 5 | 95 |
| **6.90** | 400 | 5 | 95 |
| **7.00** | 400 | 97 | 3 |
| **11.00** | 400 | 97 | 3 |

**Supplemental Table 1** LC Pump Gradient

After the analytical column a column switching valve was placed, so that only the eluate of interest between 0.5 and 9.5 min, where the analytes elute from the column, was introduced into the mass spectrometer and then analyzed in MRM mode with positive polarity. In this way, excessive contamination of the ion source can be avoided. In order to avoid carry-over from sample to sample, an additional rinsing of the needle was integrated into the method in the autosampler after each injection. The specific MRM transition for each molecule is shown in Table 2. The IonSpray Voltage (IS) was 4000 V, the Curtain Gas flow was 40.0 psi and the ion source temperature was set at 550°C.

| **Analyte** | **Q1 mass (m/z)** | | | **Q3 mass (m/z)** | | **DP (volts)** | | **CE (volts)** |  |
| --- | --- | --- | --- | --- | --- | --- | --- | --- | --- |
| **TRP** | 205.100 | | | 118.000 | | 39 | | 23 |  |
| **KYN** | 209.1 | | | 94.1 | | 41 | | 20 |  |
| **3-HKYN** | | | 225.1 | 110 | | 51 | | 25 | |
| **PICO** | | | 124.00 | 78.00 | | 51 | | 27 | |
| **KYNA** | | | 190.10 | 144.00 | | 71 | | 30 | |
| **XANA** | | | 206.00 | 178.00 | | 56 | | 22 | |
| **5-HT** | | | 177.10 | 160.10 | | 51 | | 17 | |
| **NEO** | | | 254.1 | 206.1 | | 85 | | 26 | |
| **1-MTP** | | | 219.10 | 202.10 | | 41 | | 8 | |
| **8-O-3-DOG** | | | 284.1 | 168.00 | | 71 | | 19 | |
| **3-HAA** | | | 154.00 | 136.00 | | 40 | | 17 | |
| **MEL** | | | 233.1 | 174.0 | | 50 | | 18 | |
| **QUIN** | | | 168.00 | 124.00 | | 46 | | 17 | |
| **DOPA** | | | 154.1 | 137.00 | | 41 | | 15 | |
| **d5-KYNA** | | | 195.1 | 121.00 | | 51 | | 45 | |
| **d5-PHE** | | | 171.10 | 125.10 | | 66 | | 21 | |
| **d6-KYN** | | | 215.1 | 198.1 | | 61 | | 15 | |
| **d5-TRP** | | | 210.1 | 122.1 | | 31 | | 37 | |
| **N15-8-Oxo** | | | 289.1 | 173.0 | | 71.0 | | 17.0 | |

**Supplemental Table 2** Mass transitions, de-clustering potential (DP) and collision energy (CE) for each molecule measured in this method.

For the quantification of the metabolites, internal standards were utilized. Since a deuterated internal standard was not available for all analytes examined here, the internal standards were used for 3-HKYN, PICO, XANA, 5-HT, NEO, 1-MTRP, 3-HAA, MEL, QUIN and DOPA, which were most similar in terms of their chemical structure and whose retention time was closest. The evaluation of the data, including peak integration and concentration determination, was performed with Analyst software (Version 1.5.1, AB Sciex, Darmstadt, Germany).

# Supplemental Figures

## Supplemental Figure 1

**
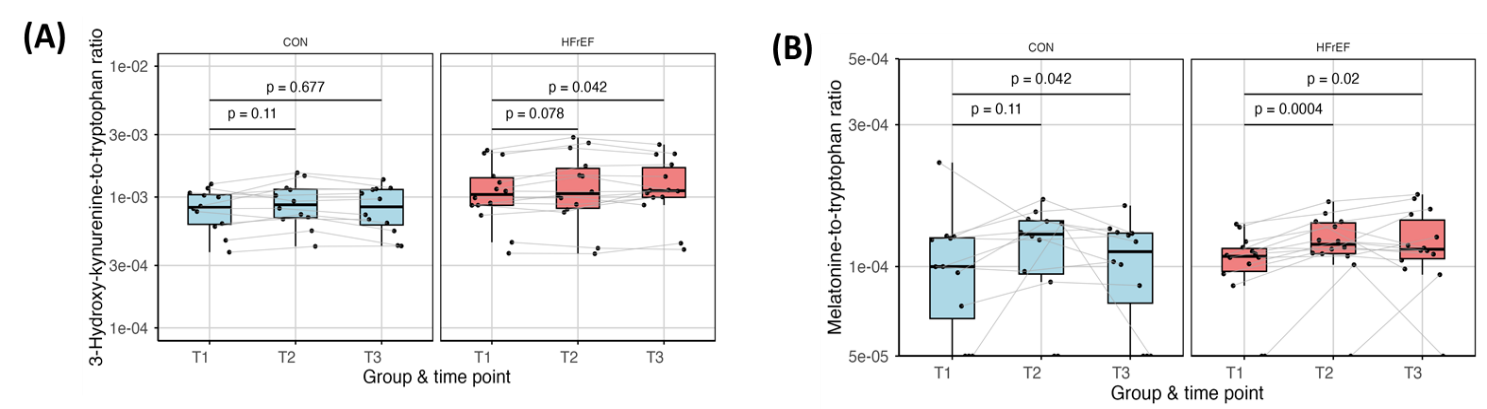
**


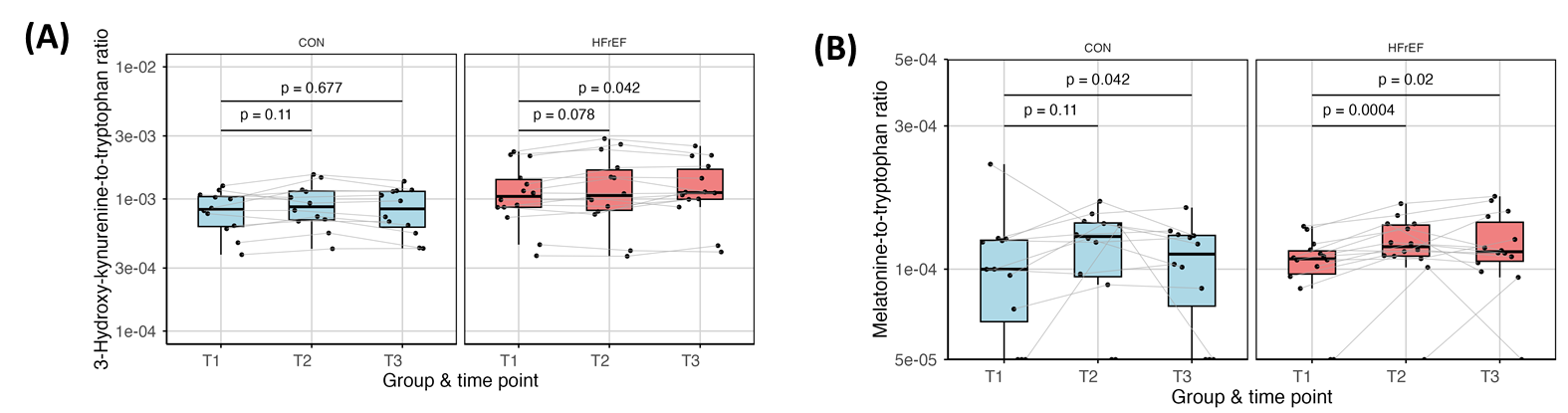

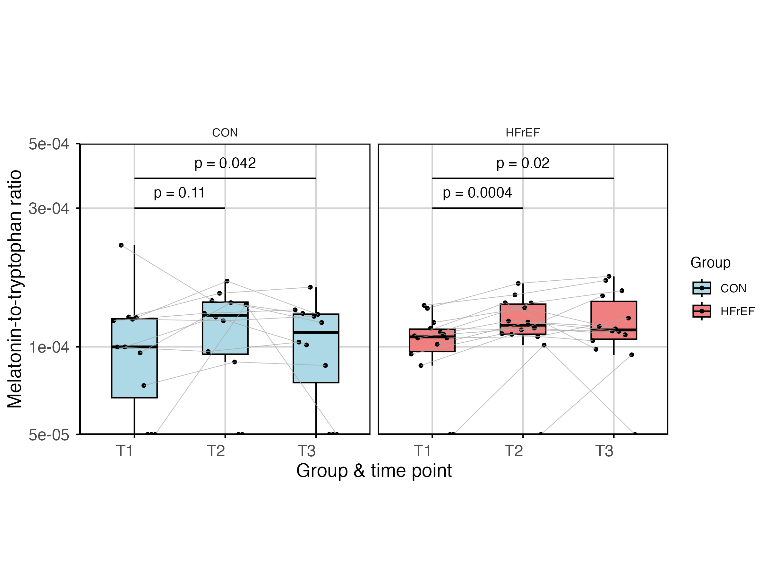


**
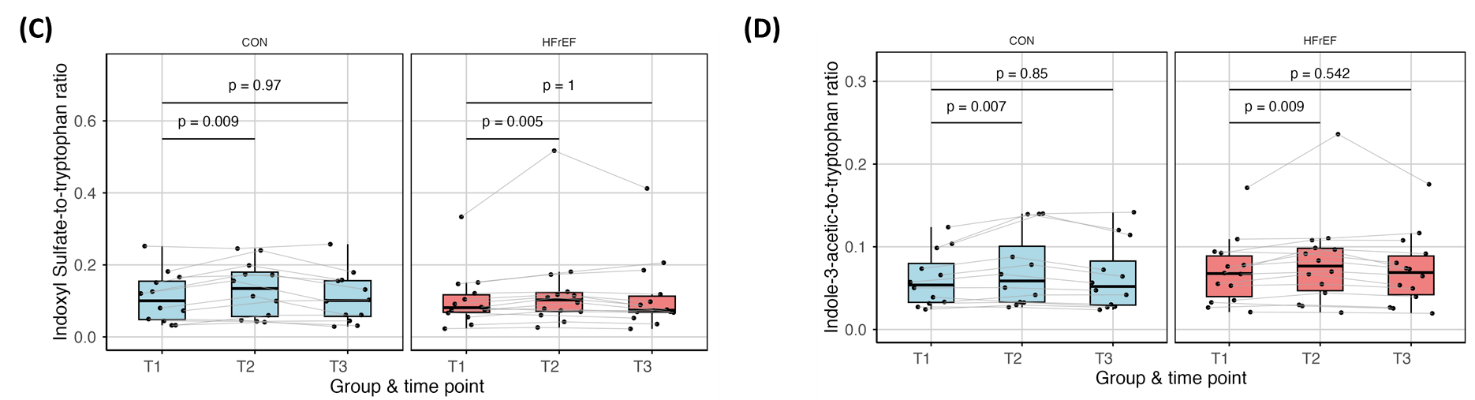
**

**
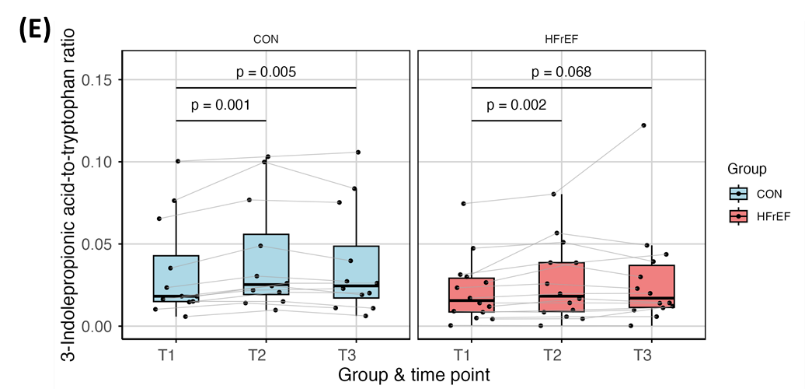
**

**Supplemental Figure 1**: Further metabolization of tryptophan into 3-hydroxy-kynurenine is delayed and more pronounced in the HFrEF group (**A**). Melatonin synthesis from TRP increases at T2 in HFrEF and T3 in both groups (**B**). Indoxyl sulphate (**C**), indole-3-acetic acid (**D**) and 3-indole propionic acid (**E**) along the indole pathway increases at T2 in both groups.

## Supplemental Figure 2

**
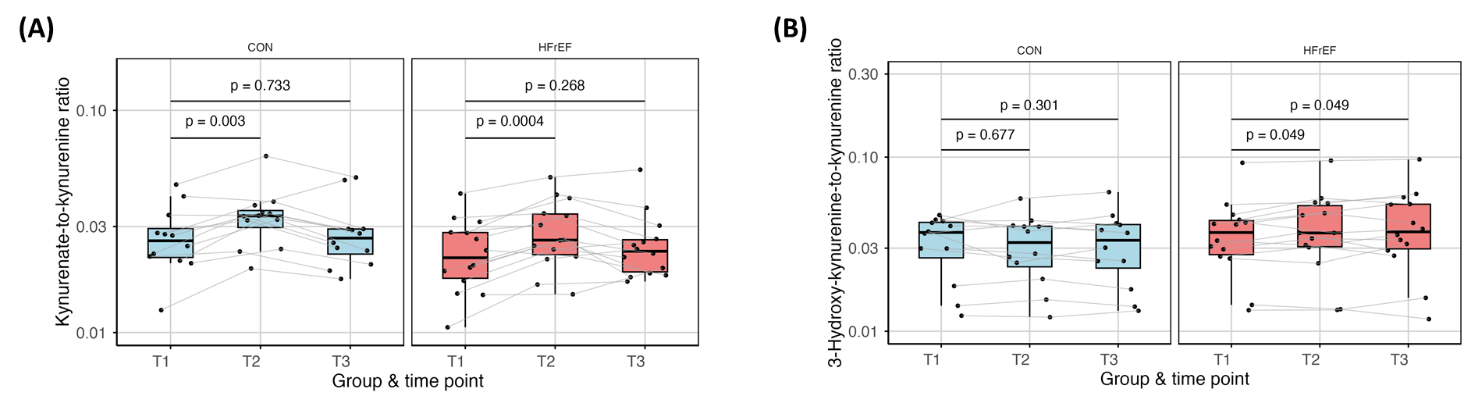
**

**
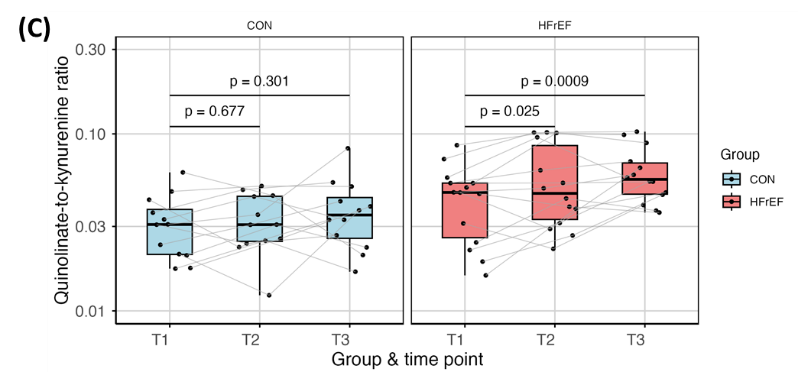
**

**Supplemental Figure 2**: Kynurenine metabolization into kynurenate increases acutely after CPET and returns to baseline after recovery in controls and HFrEF (**A**), while kynurenine metabolization into 3- hydroxy-kynurenine (**B**) and quinolinate (**C**) increases only in HFrEF, but not in controls.

## Supplemental Figure 3

| **(A)** | **(B)** |
| --- | --- |
| 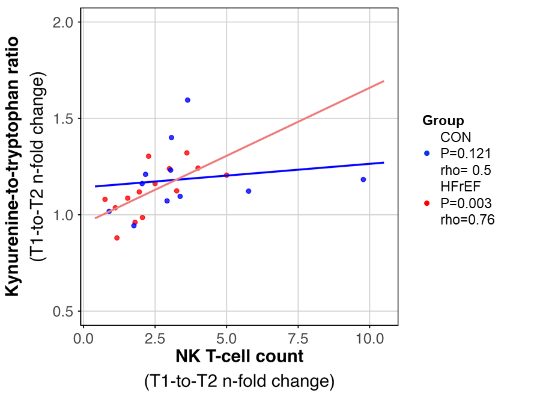 | 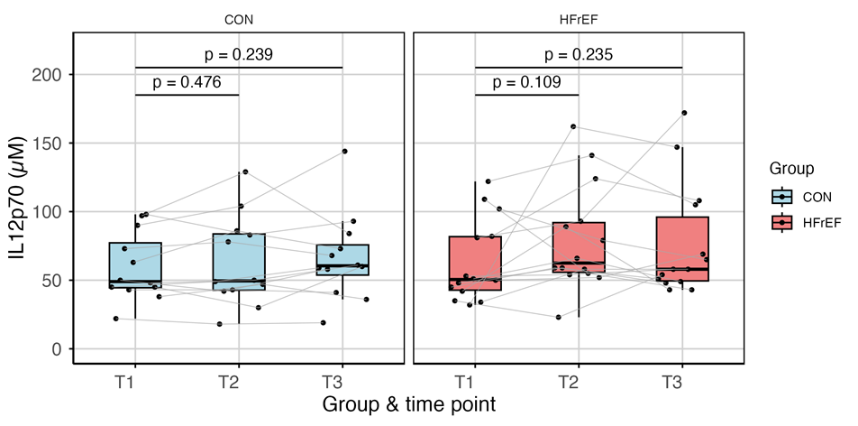 |

**Supplemental Figure 3**: Increases in NK-T cell count (**A**) between T1 and T2 correlate with increase in the kynurenine-to-tryptophan ratio during the same time in HFrEF. No significant differences were observed in the plasma IL12p70 levels between T1 and T2 in the HFrEF group and control group (**B**).

## Supplemental Figure 4

| **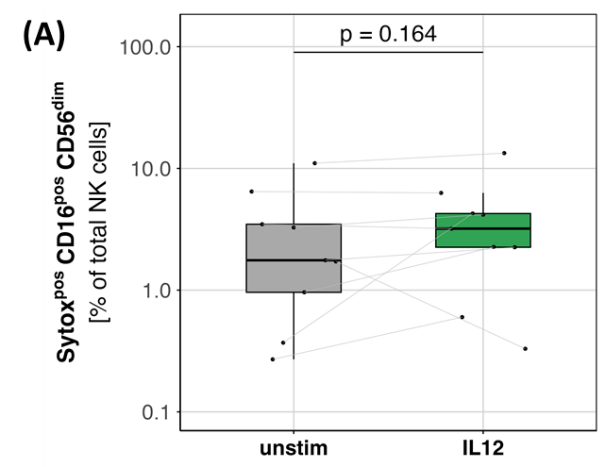** | **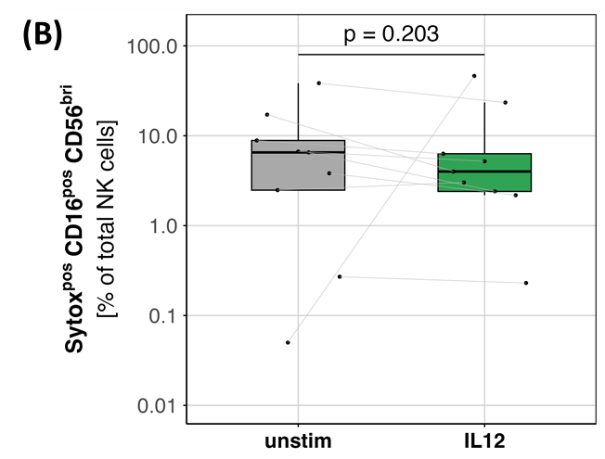** |
| --- | --- |
| **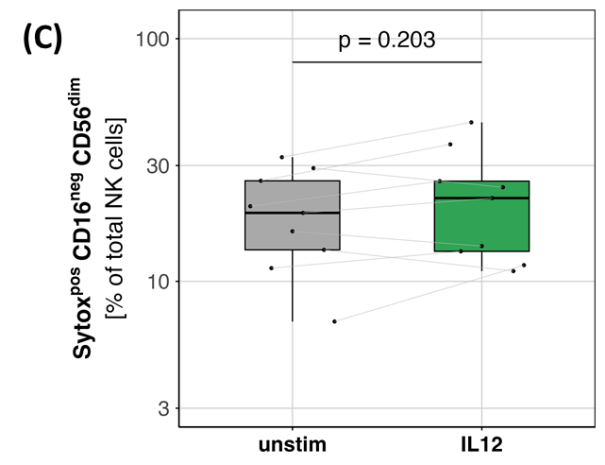** | 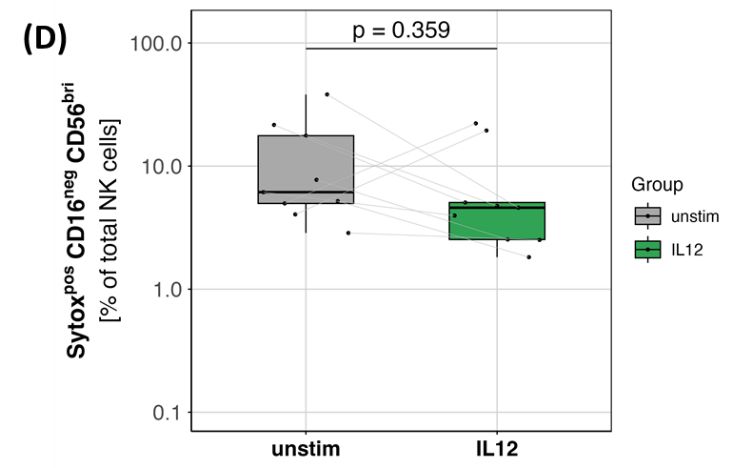 |

**Supplemental Figure 4**: Cell death rates do not differ with IL-12 treatment between NK cell phenotypes.

## Supplemental Figure 5

**
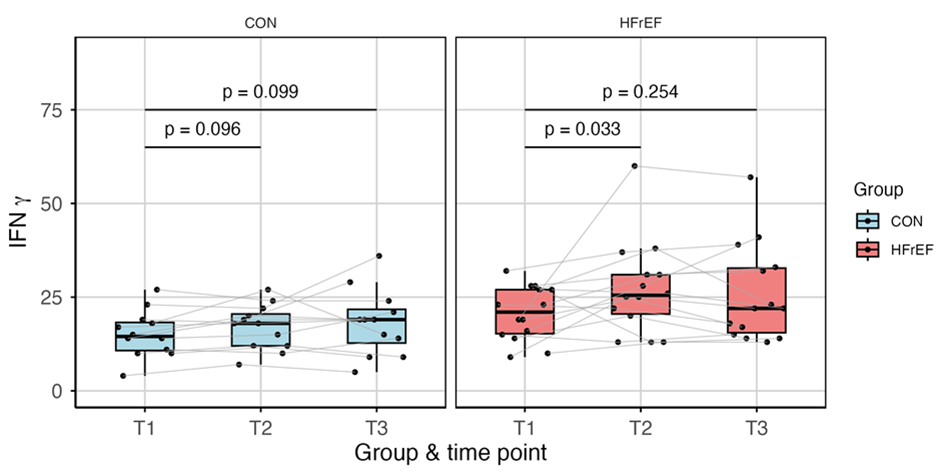
**

**Supplemental Figure 5**: IFN-γ tends to increase between T1 and T2 in both groups.

## Supplemental Figure 6


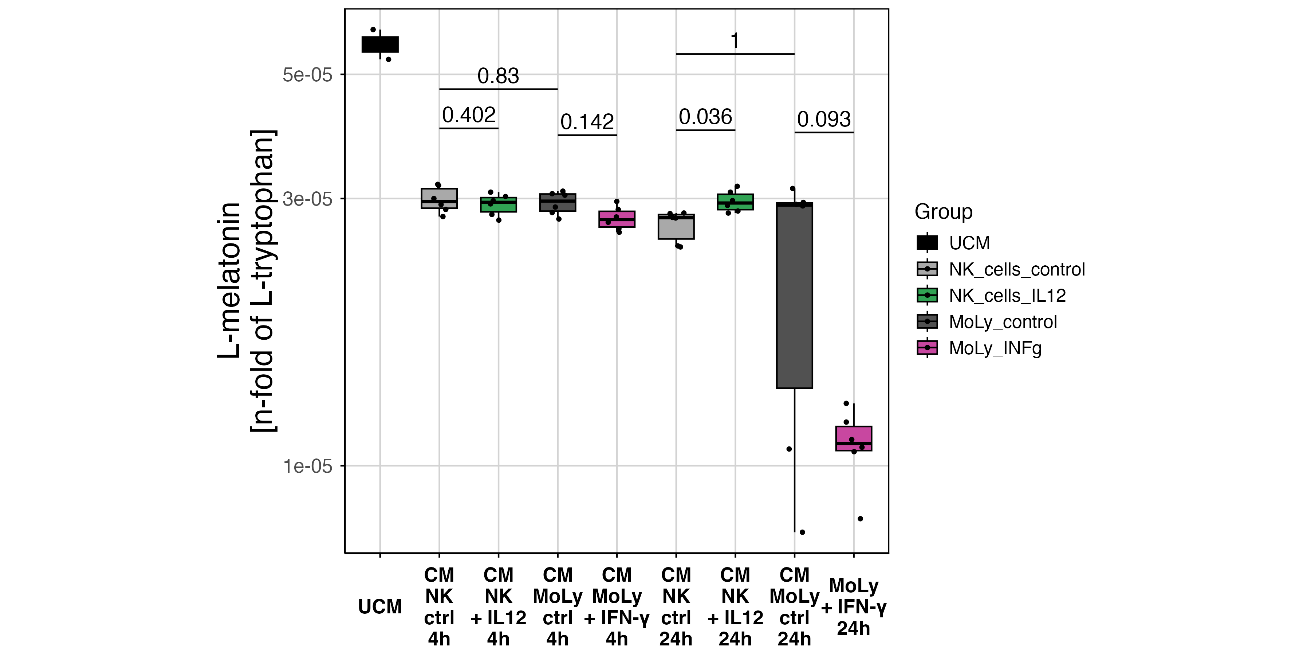

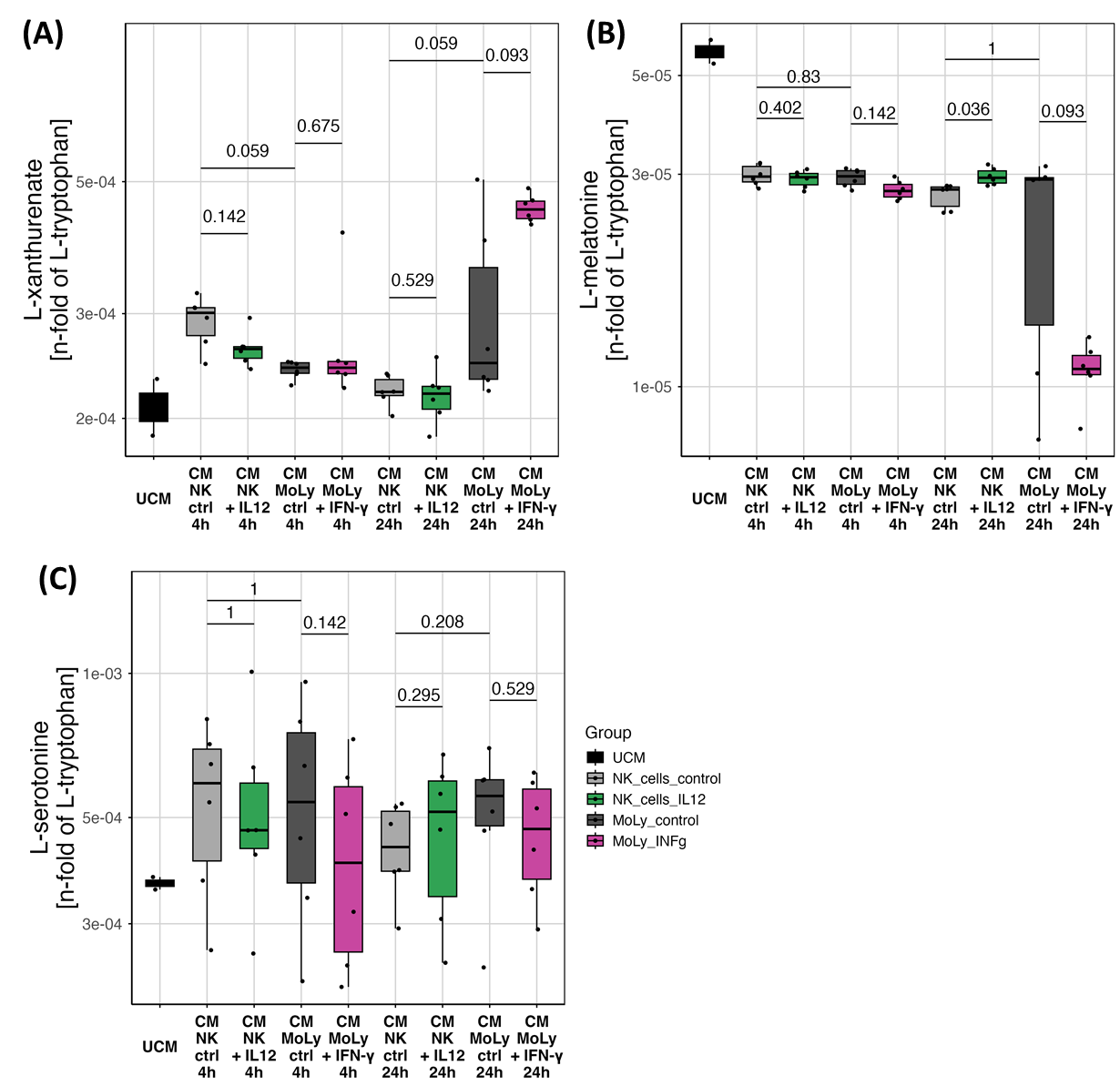


**
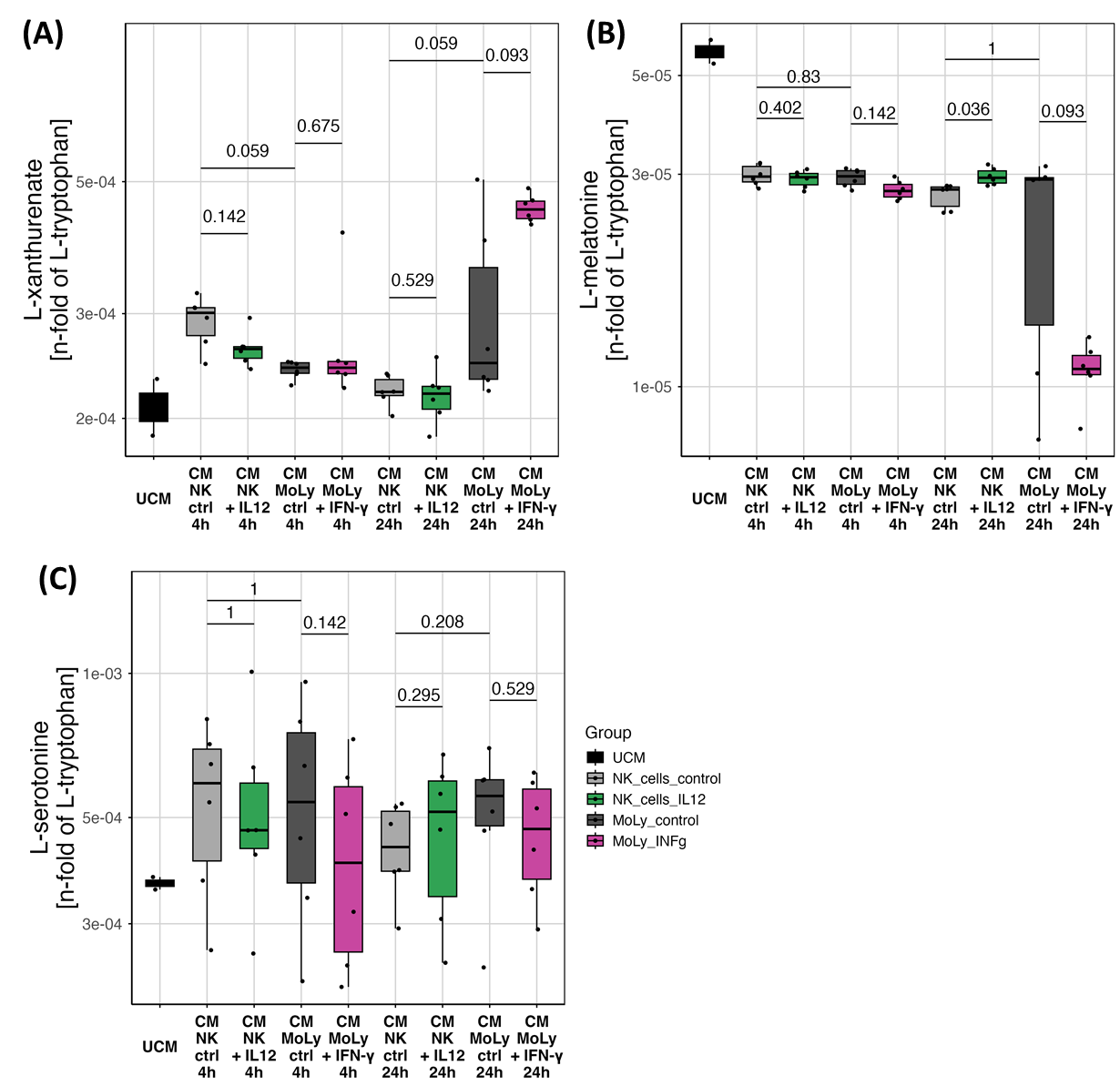
**


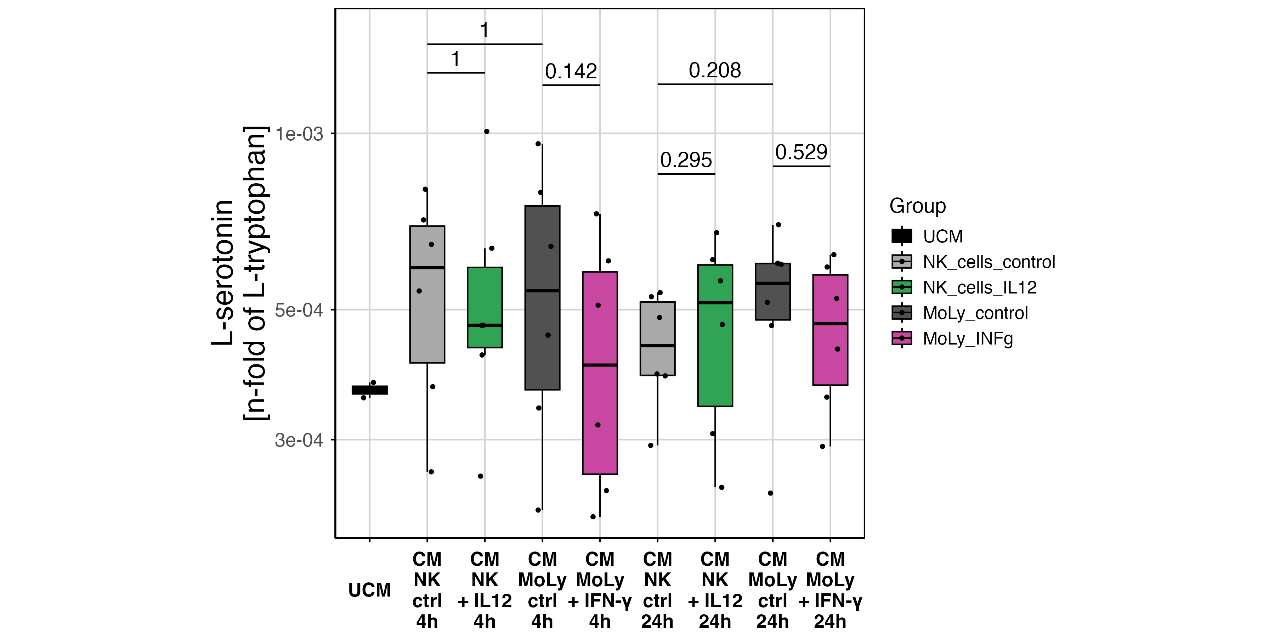


**(C)**

**Supplemental Figure 6**: Ratios of TRP metabolites versus TRP in cell culture supernatants at 4h and 24h, as well as unconditioned medium control.

## Supplemental Figure 7

**
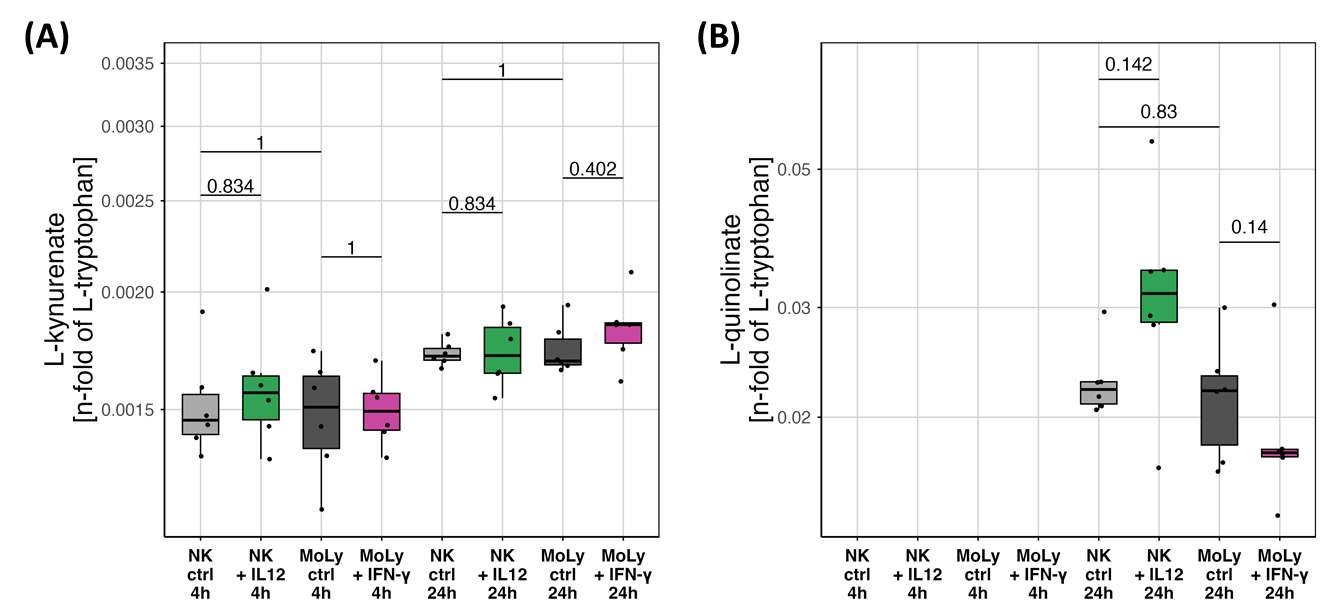
**


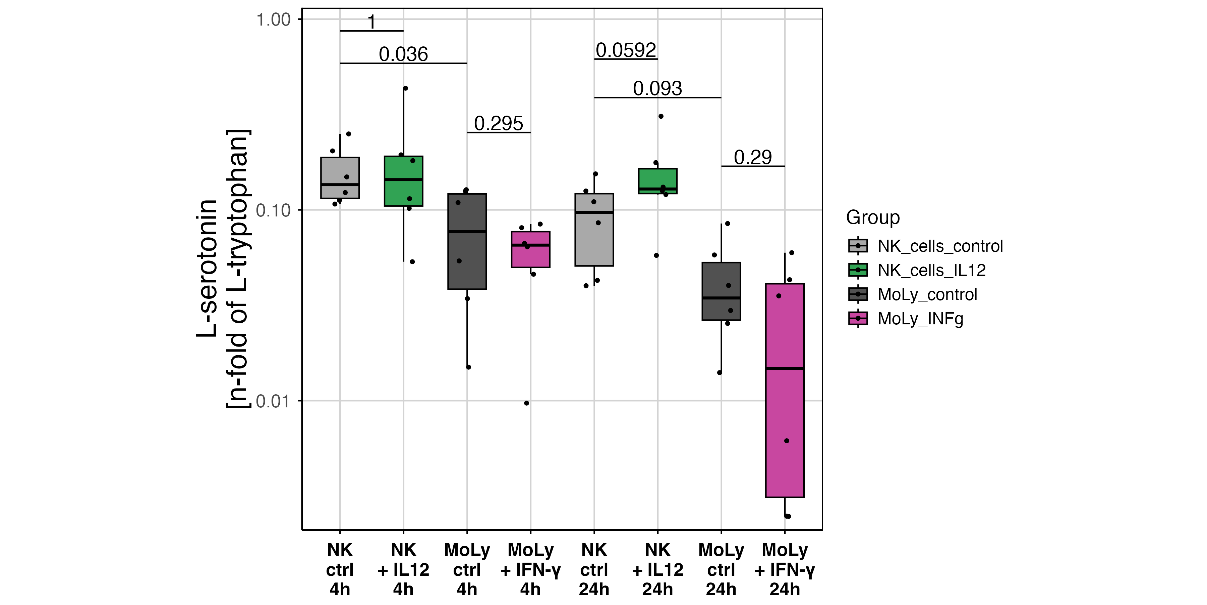


**(D)**

**
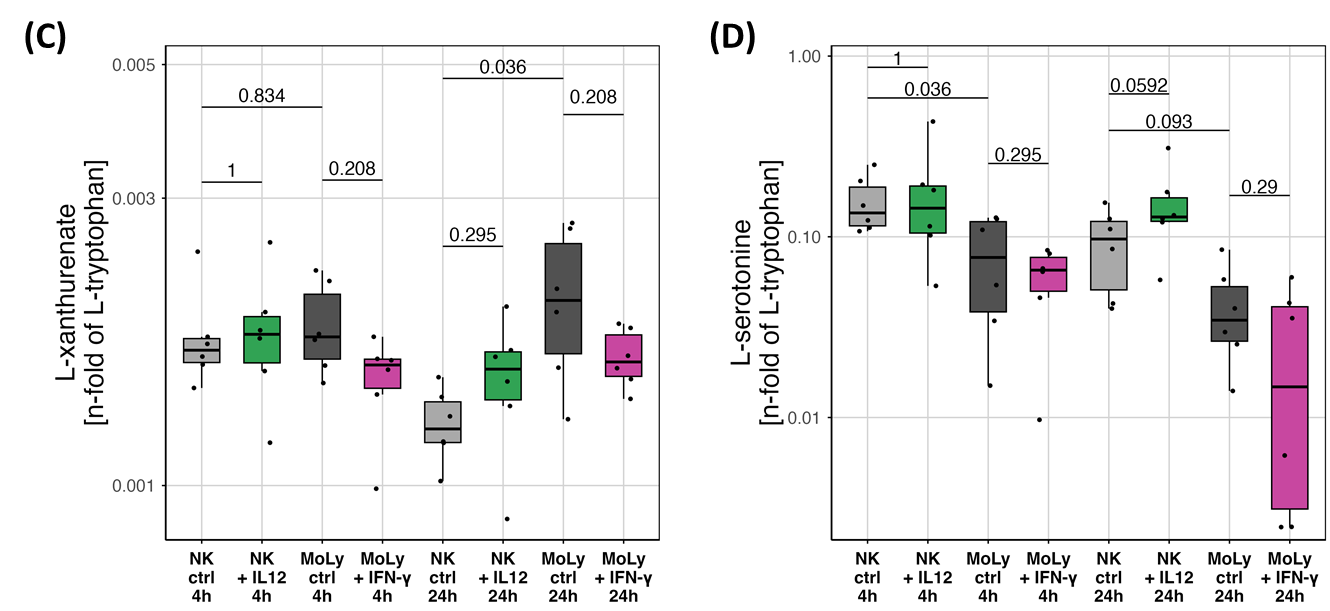
**


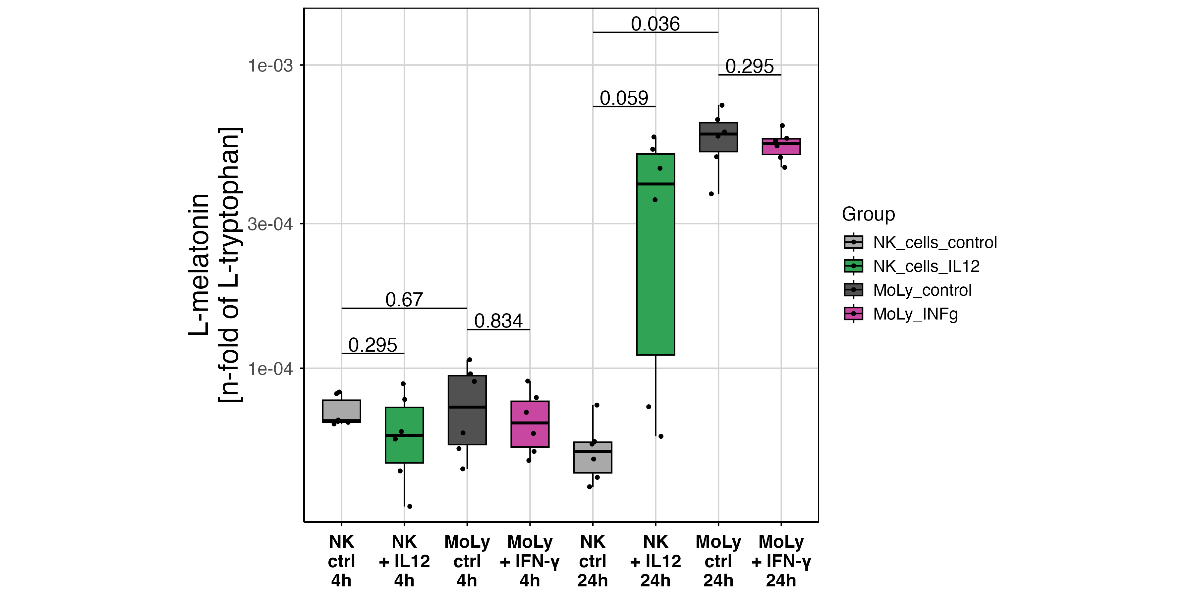


**(E)**

**Supplemental Figure 7**: Ratios of TRP metabolites versus TRP in NK cells and Mono-/Lymphocytes at 4h and 24h. In the 4h time point cell lysates, QUIN was below detection limit (too many missings).

# Supplemental Tables

**Supplemental Table I:** Correlations between the increase in the kynurenine-to-tryptophan ratio between T1 and T2 and changes in numbers of circulating leukocyte subset between the same time points.

|  | **control** | | **HFrEF** | |
| --- | --- | --- | --- | --- |
| **parameter** | **rho** | **p_adj_** | **rho** | **p_adj_** |
| **NK cells [n]** | 0.509 | 0.729 | 0.697 | **0.044** |
| **NK-T cells [n]** | 0.5 | 0.729 | 0.758 | **0.03** |
| CD14^hi^CD16^neg^ classical monocytes [n] | 0.112 | 0.817 | 0.231 | 0.512 |
| CD14^hi^CD16^pos^ intermediate monocytes [n] | 0.147 | 0.817 | 0.09 | 0.762 |
| CD14^lo^CD16^pos^ non-classical monocytes [n] | 0.147 | 0.817 | 0.108 | 0.762 |
| CD8^pos^ cytotoxic T lymphocytes [n] | 0.105 | 0.817 | 0.481 | 0.252 |
| CD4^pos^CD127^pos^CD25^lo/neg^ T_n/cm_ cells [n] | 0.098 | 0.817 | 0.521 | 0.236 |
| CD4^pos^CD127^neg^CD25^neg^ Teff cells [n] | -0.133 | 0.817 | 0.358 | 0.379 |
| CD4^pos^CD25^pos^CD127^neg^ Treg cells [n] | -0.231 | 0.817 | 0.389 | 0.379 |
| neutrophil granulocytes [n] | 0.077 | 0.817 | 0.284 | 0.434 |

**Supplemental Table II:** Correlations between the increase in the kynurenine-to-tryptophan ratio between T1 and T2 and changes in plasma cytokine levels between the same time points.

|  | **control** | | **HFrEF** | |
| --- | --- | --- | --- | --- |
| **parameter** | **rho** | **p_adj_** | **rho** | **p_adj_** |
| E selectin | 0.133 | 0.96 | -0.578 | 0.109 |
| GM-CSF | 0.056 | 0.96 | 0.468 | 0.149 |
| IFN-alpha | 0.017 | 0.96 | 0.42 | 0.184 |
| IFN-gamma | -0.256 | 0.948 | 0.442 | 0.169 |
| **IL-10** | -0.345 | 0.948 | 0.853 | **0.001** |
| **IL-12p70** | 0.273 | 0.948 | 0.899 | **<0.0001** |
| IL-17A | -0.392 | 0.948 | 0.565 | 0.109 |
| IL-1 alpha | 0.161 | 0.96 | 0.657 | 0.051 |
| **IL-1 beta** | -0.238 | 0.948 | 0.771 | **0.012** |
| IL-4 | 0.315 | 0.948 | 0.512 | 0.149 |
| IL-6 | 0.217 | 0.948 | 0.49 | 0.149 |
| IL-8 | 0.345 | 0.948 | 0.485 | 0.149 |
| IP-10 | -0.105 | 0.96 | 0.16 | 0.616 |
| MCP-1 | -0.403 | 0.948 | 0.56 | 0.109 |
| MIP-1 alpha | 0.084 | 0.96 | 0.474 | 0.149 |
| MIP-1 beta | -0.462 | 0.948 | 0.235 | 0.467 |
| P selectin | 0.077 | 0.96 | 0.402 | 0.197 |
| sICAM-1 | 0.035 | 0.96 | -0.064 | 0.832 |
| TNF-alpha | -0.091 | 0.96 | 0.275 | 0.405 |

**Supplemental Table III:** Correlations between the increase in circulating NK cell numbers between T1 and T2 and changes in plasma cytokine levels between the same time points.

|  | **control** | | **HFrEF** | |
| --- | --- | --- | --- | --- |
| **parameter** | **rho** | **p_adj_** | **rho** | **p_adj_** |
| E selectin | -0.173 | 0.945 | -0.213 | 0.515 |
| **GM-CSF** | -0.082 | 0.989 | 0.749 | **0.028** |
| IFN-alpha | -0.052 | 0.989 | 0.391 | 0.209 |
| IFN-gamma | 0.351 | 0.731 | 0.402 | 0.207 |
| IL-10 | 0.009 | 0.989 | 0.591 | 0.058 |
| **IL-12p70** | 0.309 | 0.731 | 0.675 | **0.035** |
| IL-17A | 0.336 | 0.731 | 0.515 | 0.099 |
| **IL-1 alpha** | 0.121 | 0.965 | 0.754 | **0.028** |
| **IL-1 beta** | 0.136 | 0.965 | 0.662 | **0.035** |
| **IL-4** | 0.473 | 0.731 | 0.732 | **0.028** |
| IL-6 | 0.491 | 0.731 | 0.235 | 0.491 |
| **IL-8** | 0.019 | 0.989 | 0.652 | **0.035** |
| IP-10 | -0.282 | 0.731 | 0.446 | 0.16 |
| MCP-1 | -0.3 | 0.731 | 0.16 | 0.615 |
| MIP-1 alpha | -0.018 | 0.989 | 0.558 | 0.076 |
| MIP-1 beta | -0.309 | 0.731 | 0.481 | 0.129 |
| P selectin | 0.182 | 0.945 | 0.543 | 0.087 |
| sICAM-1 | -0.3 | 0.731 | -0.033 | 0.916 |
| TNF-alpha | 0.309 | 0.731 | 0.604 | 0.058 |
